# Tilianin Extracted From *Dracocephalum moldavica* L. Induces Intrinsic Apoptosis and Drives Inflammatory Microenvironment Response on Pharyngeal Squamous Carcinoma Cells via Regulating TLR4 Signaling Pathways

**DOI:** 10.3389/fphar.2020.00205

**Published:** 2020-03-04

**Authors:** Hailun Jiang, Li Zeng, Xueqi Dong, Shuilong Guo, Jianguo Xing, Zhuorong Li, Rui Liu

**Affiliations:** ^1^Institute of Medicinal Biotechnology, Chinese Academy of Medical Sciences and Peking Union Medical College, Beijing, China; ^2^Department of Cardiology, Fuwai Hospital, National Center for Cardiovascular Disease, Chinese Academy of Medical Sciences and Peking Union Medical College, Beijing, China; ^3^Department of Gastroenterology, Beijing Friendship Hospital, Capital Medical University, Beijing, China; ^4^Xinjiang Institute of Materia Medica, Ürümqi, China

**Keywords:** dendritic cells, human pharyngeal squamous cell carcinoma, intrinsic apoptosis, nuclear factor-κappa B, tilianin, toll-like receptor, tumor immunity

## Abstract

Human pharyngeal squamous cell carcinoma is highly invasive and proliferative, and exhibits an extremely low 5-year survival rate due to poor understanding of the underlying pathogenic mechanisms, and lack of efficient treatment. It has been shown that the immunosuppressive microenvironment created by tumor cells impairs the immune response against tumor progression, thereby affecting the prognosis for tumor patients. Thus, to improve therapeutic efficacy, it is critical to identify novel drugs with immunoinflammatory modulatory properties to treat tumor immune evasion. Tilianin, the main ingredient of total flavonoids extracted from *Dracocephalum moldavica* L., has multiple biological functions, including cardiovascular protective effects, anti-tumor effects, and anti-inflammatory effects. In the present study, the suppressive effects of tilianin on human pharyngeal squamous cell carcinoma were investigated and the underlying mechanisms in regulating the tumor immunosuppressive microenvironment were explored. The cytotoxicity of tilianin on FaDu cells was determined by CCK-8 and clone formation assays. Moreover, the levels of toll-like receptor 4 (TLR4) signaling transduction and apoptotic pathways were determined by immunocytochemical, biochemical, and molecular biological technologies. In addition, the maturation of dendritic cells (DCs) that were co-cultured in supernatant of FaDu cells was evaluated by flow cytometry to investigate alterations in immune system function. For mechanistic exploration, TLR4 siRNA, p38 siRNA, c-Jun N-terminal kinase (JNK) siRNA, and p65 siRNA were used as loss-of-function target evaluation of tilianin therapy. Combined, these results showed that tilianin treatment increased cytotoxicity as well as the apoptotic population of FaDu cells in a dose-dependent manner. Furthermore, tilianin treatment decreased the level of anti-apoptotic markers Bcl-2 and Bcl-xL, increased the level of apoptotic factors Bad and Bax, and stimulated cytochrome *c* release, caspase-3 and poly ADP ribose polymerase (PARP) activation in FaDu cells. Furthermore, our findings indicated that tilianin treatment activated TLR4/p38/JNK/NF-κB signaling pathways and increased the release of inflammatory cytokines. This promoted the maturation of DCs to enhance immune system function in the tumor microenvironment. Moreover, the effects of tilianin on immune system function were abolished by TLR4 siRNA and p65 siRNA. In conclusion, these findings suggested that tilianin may be of immunotherapeutic value for inhibiting human pharyngeal squamous cell carcinoma.

## Introduction

Head and neck squamous cell carcinoma (HNSCC) is the sixth most common cancer worldwide, and is associated with a high mortality rate and invasion (Horton et al., 2019). Smoking and alcohol consumption are the main risk factors for HNSCC (Sturgis and Cinciripini, 2007; Pelucchi et al., 2008). Human pharyngeal squamous cell carcinoma is part of HNSCC. Cancers of the squamous cells of the head and neck are often considered together because of the many similarities they share (Cooper et al., 2009). Although clinical interventions, including surgery, radiotherapy, and chemotherapy have improved, the prognosis of HNSCC is poor when compared with other solid tumors, and the overall 5-year survival rate is very low (Lacko et al., 2014; Allen et al., 2015). In previous studies, it has been demonstrated that the immunosuppressive microenvironment destroys the immune response to tumors, thereby affecting the prognosis of cancer patients (Davis et al., 2016). Therefore, to improve therapeutic efficacy, it is essential to identify novel drugs for the treatment of pharyngeal squamous cell carcinoma by destroying the immunosuppressive environment produced by tumor cells.

Toll-like receptors (TLRs) are a family of receptor molecules that are predominantly present on mammalian cell surfaces. TLRs were highly conserved in evolution, and primarily recognize pathogen-associated molecular pattern (PAMPs) to rapidly activate downstream cascades, promote the secretion of cytokines and chemical factors, and ultimately activate natural and adaptive immune responses to eliminate pathogens (Yu et al., 2013). TLR4, a member of the TLR family, has been intensively studied, and plays an important role in tumor surveillance and regulation (Núñez et al., 2012). During tumorigenesis, endogenous ligands bind TLR4, resulting in downstream inflammatory reactions and the recruitment of inflammatory cells, including neutrophils and dendritic cells (DCs), and induce the body’s immune response to tumors (Apetoh et al., 2007). Conversely, if TLR4 is inactivated or deleted, the immune system loses its ability to respond to the tumor, and enters the immune escape mechanism of the tumor (Yusuf et al., 2008; Núñez et al., 2012). In a previous study, it was shown that TLR4 plays an important role in the molecular mechanism of *Salmonella choleraesuis* (*S. choleraesuis*)-induced host anti-tumor responses (Lee et al., 2008).

*Dracocephalum moldavica* L. (*D. moldavica*), a member of the Labiatae family, is distributed in several provinces of Northeast China, Northwest China, and North China. Southeastern Xinjiang is rich in *D. moldavica*, which is used as a popular herb in traditional Uygur folk medicine and serves as a single medicine prescription in the Uyghur Medicine Ministerial Standard. *D. moldavica* is mainly used for the treatment of a variety of cardiovascular diseases (Guo et al., 2015; Jia et al., 2017; Tan et al., 2017; Shen et al., 2019). Modern pharmacological studies have illustrated that the active ingredients in *D. moldavica* displayed the ability to prevent or treat neurodegenerative disorders and inflammatory disorders (García-Díaz et al., 2016; Liu et al., 2018), and suppressed the growth and proliferation of various types of cancer cells (Sato et al., 2015; Chakrabarti and Ray, 2016). Tilianin is the major effective component of the total flavonoid extract from *D. moldavica* (Zeng et al., 2016). Tilianin has been reported to have neuroprotective and cardioprotective effects in the treatment of cardiovascular and cerebrovascular diseases (Zeng et al., 2018; Jiang et al., 2019). Moreover, in previous studies, it was reported that tilianin displayed anti-tumor effects in human lung adenocarcinoma and anti-angiogenesis effects based on VEGF-A (Meng, 2018; Meng et al., 2018). However, potential therapeutic effects and the underlying mechanisms of action of tilianin on pharyngeal squamous cell carcinoma have not yet been elucidated.

The current study was designed to investigate the growth inhibitory effect of tilianin on pharyngeal squamous carcinoma cell line FaDu and to explore the potential mechanism for inhibiting cell proliferation, inducing apoptosis, and stimulating DC maturation.

## Materials and Methods

### Reagents

**FIGURE 1:**
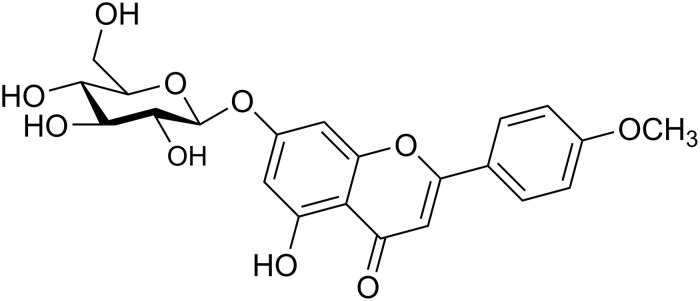
Chemical structure of tilianin. The molecular formula of tilianin is C_22_H_22_O_10_.

Tilianin (Figure 1) is a single compound extracted from *D. moldavica* by Xinjiang Institute of Materia Medica (Ürümqi, China). TLR4 siRNA, p65 siRNA, p38 MAPK siRNA, c-Jun N-terminal kinase (JNK) siRNA, and corresponding negative controls (NCs) were purchased from Santa Cruz (Dallas, TX, United States). Lipofectamine 2000 reagent (Thermo Fisher Scientific, Carlsbad, CA, United States) was used for the transfection of siRNA at a final concentration of 50 nM. Lipopolysaccharide (LPS) and human recombinant tumor necrosis factor alpha (TNF-α) were purchased from Sigma-Aldrich (Merck KGaA, Darmstadt, Germany) and Proteintech (Rosemont, IL, United States), respectively.

### Plant Materials

Whole plants of *D. moldavica* were collected in Jimusaer, Xinjiang, in July 2017 (batch number: 20170713), and identified by Prof. Jiang He, Xinjiang Institute of Materia Medica (Ürümqi, China). A voucher specimen (D170713) was deposited in the Medicinal Herbarium of Xinjiang Institute of Materia Medica (Ürümqi, China).

### Extraction and Isolation of Tilianin

The aboveground parts from *D. moldavica* (90 kg) were air-dried and powdered at room temperature (RT), then refluxed three times with 40% EtOH at 100°C. The combined EtOH solution was filtered and evaporated under reduced pressure to yield a crude extract (3.2 kg), which was partitioned using column chromatography with HPD600 resin and eluted with water, 50% EtOH and 70% EtOH. To remove impurities, the 70% EtOH eluent was filtered on a silica gel column (100–200 mesh, chloroform: methanol, 95:5–90:10–80:20). The purified product was collected. The structure of the compound was determined by its physico-chemical and spectral data (LC–MS, 1D and 2D NMR), which agreed with those reported in the literature (Tan et al., 2017). A total of 280 mg of tilianin was obtained and the purity of the compound was 99% as determined by high performance liquid chromatography (HPLC) (Supplementary Figure 1).

### Cell Culture and Treatment

Human pharyngeal squamous carcinoma cells (FaDu cells) were purchased from the American Type Culture Collection (ATCC, Manassas, VA, United States). In brief, FaDu cells were maintained in minimum essential medium (MEM, Gibco, Grand Island, NY, United States) supplemented with 10% fetal bovine serum (FBS, Gibco) in a humidified incubator at 37°C and 5% CO_2_.

FaDu cells were seeded in 6-well plates at a density of 1 × 10^5^ cells/mL, followed by subsequent experiments after overnight culture. For pharmacodynamic studies, FaDu cells were divided into four groups according to tilianin concentrations (0, 10, 30, and 100 μM), and incubated for 24 h for apoptosis assay, and 48 h for qPCR, Western blot, and ELISA analyses, and 14 days for the plate clone formation assay. For target confirmation studies, FaDu cells were randomly divided into three groups: NC siRNA transfection group, TLR4 siRNA transfection group, and the p65 transfection group. Each group was further divided into four subgroups based on the concentrations of tilianin (0, 10, 30, and 100 μM). Tilianin treatment was performed 12 h after transfection with siRNAs.

### Evaluation of Tilianin for *in vitro* Tumor Cell Killing

The growth inhibitory effect of tilianin on FaDu cells was determined by the cell counting kit-8 (CCK-8, Vazyme Biotech, Nanjing, China). In brief, FaDu cells were seeded in 96-well plates at a density of 1 × 10^4^ cells/mL (1 × 10^3^ cells/well). After culturing overnight, tilianin at a final concentration of 0, 3, 5, 10, 20, 30, 50, or 100 μM was added and co-cultured with cells for 72 h. In addition, transfection with siRNAs (NC, TLR4, p65, p38, JNK) was performed 12 h before tilianin treatment in the target confirmation test. Subsequently, 10 μL of CCK-8 reagent was added to each well for 2 h, then the cell viability was determined. The absorbance was read at 450 nm using a SPARK 20M (TECAN, Männedorf, Switzerland). Results were expressed as cell growth inhibition rates at various concentrations of tilianin.

### Cell Apoptosis Assay by Flow Cytometer

To determine cell apoptosis of FaDu cells after transfection with different siRNAs and treatment with different concentrations of tilianin (0, 10, 30, 100 μM), the fluorescein isothiocyanate (FITC)-Annexin V/propidium iodide (PI) apoptosis detection kit (Dojindo Laboratory, Kumamoto, Japan) was used. In brief, cells were resuspended in binding buffer, and stained with a mixture of FITC-labeled Annexin-V and PI for 15 min at RT and protected from light. Then, cells were analyzed by a FACSCanto II flow cytometer (BD Biosciences, New York, NY, United States). The apoptotic rate was calculated and expressed as the ratio of FITC-Annexin V and PI-positive cells to the total number of cells.

### Plate Clone Formation Assay

FaDu cells were seeded in 6-well plates at a density of 200 cells per well, and incubated for 6 h to allow the cells to adhere to the plate. Next, cells were transfected with siRNA and tilianin. Cells were cultured for 14 days at 37°C. Cells were washed with PBS and fixed with paraformaldehyde (PFA) for 20 min at RT. Then, cells were stained with crystal violet (Beyotime, Shanghai, China) for 15 min at RT, and rinsed with water. Cells were air dried for at least 1 h at RT, after which colonies were photographed by a Fusion-FX6 imaging system (Vilber Lourmat, Marne-la-Vallée, France). Clone formation was calculated using Image J. software (National Institutes of Health, Bethesda, MD, United States).

### Dendritic Cell Isolation and the Detection of Cell Surface Markers by Flow Cytometry

Human monocyte-derived immature DCs were obtained from peripheral blood mononuclear cells (PBMC) extracted from healthy human ethylene diamine tetraacetic acid (EDTA) anticoagulated whole blood. PBMC were extracted using Ficoll (GE, Boston, MA, United States) and seeded at a density of 5 × 10^5^ cells/mL in 24-well plates. For the induction of cells, human granulocyte-macrophage colony-stimulating factor (GM-CSF, 100 ng/mL, Proteintech) and human interleukin-4 (IL-4, 50 ng/mL, Proteintech) were added to each well. On day 4, the cell culture supernatant of each group was co-cultured with immature DCs. On day 8, positive control groups were treated with 10 μg/mL LPS or 50 ng/mL TNF-α for 24 h.

On day 9, cell surface markers of DCs were evaluated by flow cytometry. DCs were stained on ice for 30 min with FITC-conjugated anti-CD11c and phycoerythrin (PE)-conjugated anti-CD83 (Biolegend, San Diego, CA, United States), and data were acquired and analyzed on a FACSCanto II flow cytometer (BD Biosciences).

### Quantitative Reverse Transcription Polymerase Chain Reaction Analysis

Total RNA of FaDu cells was extracted using the Cell/Tissue Total RNA Isolation Mini kit (Vazyme Biotech) according to the manufacturer’s instructions. A total of 2 ng of total RNA was reverse transcribed into complementary DNA (cDNA) using the HiScript II 1st Strand cDNA Synthesis Kit (Vazyme Biotech). Quantitative polymerase chain reaction (qPCR) analysis was performed on a Real-Time PCR System (BIOER, Hangzhou, China) using ChamQ SYBR Master Mix (Vazyme Biotech). Primers used are shown in Table 1. Experiments were performed in triplicate. GAPDH served as an internal reference. The thermo cycle conditions were as follows: denaturation at 95°C for 30 s, followed by 40 cycles of denaturation at 95°C for 10 s, and extension at 60°C for 30 s. Data were analyzed using the 2^–ΔΔCT^ method.

**TABLE 1:** PCR primer sequences.

Primer name	Primer sequence
TLR4-F	5′-AGTTGATCTACCAAGCCTTGAGT-3′
TLR4-R	5′-GCTGGTTGTCCCAAAATCACTTT-3′
BAX-F	5′-CCCGAGAGGTCTTTTTCCGAG-3′
BAX-R	5′-CCAGCCCATGATGGTTCTGAT-3′
BCL2-F	5′-GGTGGGGTCATGTGTGTGG-3′
BCL2-R	5′-CGGTTCAGGTACTCAGTCATCC-3′
BAD-F	5′-CCCAGAGTTTGAGCCGAGTG-3′
BAD-R	5′-CCCATCCCTTCGTCGTCCT-3′
BCL2L1-F	5′-GAGCTGGTGGTTGACTTTCTC-3′
BCL2L1-R	5′-TCCATCTCCGATTCAGTCCCT-3′
CYCS-F	5′-CTTTGGGCGGAAGACAGGTC-3′
CYCS-R	5′-TTATTGGCGGCTGTGTAAGAG-3′
GAPDH-F	5′-ACAACTTTGGTATCGTGGAAGG-3′
GAPDH-R	5′-GCCATCACGCCACAGTTTC-3′

*F, forward; R, reverse.*

### Western Blot Analysis

Changes in protein expression levels in FaDu cells were determined by Western blot analysis. For protein extraction, the M-PER mammalian protein extraction reagent (Thermo Fisher Scientific) was applied. A total of 20 μg of protein was separated using 10% sodium dodecyl sulfate-polyacrylamide gel electrophoresis (SDS-PAGE) at 80 V for 120 min. Then, proteins were transferred onto polyvinylidene difluoride (PVDF) membranes (Millipore, Burlington, MA, United States) at 390 mA for 60 min. Membranes were blocked with 5% non-fat milk dissolved in Tris-buffered saline, containing 0.1% Tween-20 (TBST) for 1 h at RT, followed by incubation with primary antibodies (Table 2) at 4°C overnight. Subsequently, membranes were washed three times in TBST, followed by incubation with horseradish peroxidase (HRP)-conjugated goat anti-rabbit or anti-mouse IgG secondary antibody (1:10000, EarthOx Life Sciences, Millbrae, CA, United States) for 1 h at RT, then washed three times in TBST. Finally, proteins were visualized by chemiluminescent detection using an ECL detection kit (Thermo Fisher Scientific). Images and the density of protein bands were acquired on a Fusion-FX6 imaging system (Vilber Lourmat). GAPDH served as an internal control.

**TABLE 2:** Primary antibodies used in this study.

Antibody	Type	Diluted	Source
Anti-TLR4	Polyclonal	1:500	Proteintech
Anti-phospho-NF-κB p65 (Ser536)	Monoclonal	1:1000	CST
Anti- NF-κB p65	Polyclonal	1:1000	Abcam
Anti-phospho-p38 MAPK (Thr180/Tyr182)	Monoclonal	1:1000	CST
Anti-p38 MAPK	Monoclonal	1:1000	Abcam
Anti-phospho-SAPK/JNK (Thr183/Tyr185)	Monoclonal	1:2000	CST
Anti-JNK	Monoclonal	1:2000	Abcam
Anti-IκB	Monoclonal	1:1000	Abcam
Anti-phospho-IKKα/β(Ser176/180)	Monoclonal	1:1000	CST
Anti-IKKα/β	Monoclonal	1:1000	Abcam
Anti-BAX	Monoclonal	1:1000	Abcam
Anti-Bcl-2	Monoclonal	1:1000	Abcam
Anti-BAD	Polyclonal	1:500	Proteintech
Anti-Bcl-xL	Polyclonal	1:500	Proteintech
Anti-GAPDH	Polyclonal	1:5000	Proteintech

*CST, Cell Signaling Technology, Danvers, MA, United States; Abcam, Cambridge, MA, United States.*

### Caspase-3 Activity Assay

Caspase-3 activity in FaDu cells was determined by a Human Cleaved Caspase-3 (Asp175) SimpleStep ELISA kit (Abcam, Cambridge, MA, United States) according to the manufacturer’s instructions.

### ELISA Assays for TNF-α and IL-6

The levels of TNF-α and interleukin-6 (IL-6) in the supernatant of FaDu cell were measured by commercial ELISA kits according to the manufacturer’s instructions (Proteintech). Data were represented as pg/mL.

### Statistical Analysis

Data were expressed as the mean ± standard deviation (SD) and *P* < 0.05 was considered statistically significant. Statistical analysis was performed using GraphPad Prism version 8.0 (GraphPad, Inc., La Jolla, CA, United States). All experiments were performed at last in triplicate. Data were analyzed using the Student’s *t*-test or one-way ANOVA, followed by Tukey’s *post hoc* tests where appropriate.

## Results

### Tilianin Inhibited Cell Proliferation and Induced Cell Apoptosis in FaDu Cells

**FIGURE 2:**
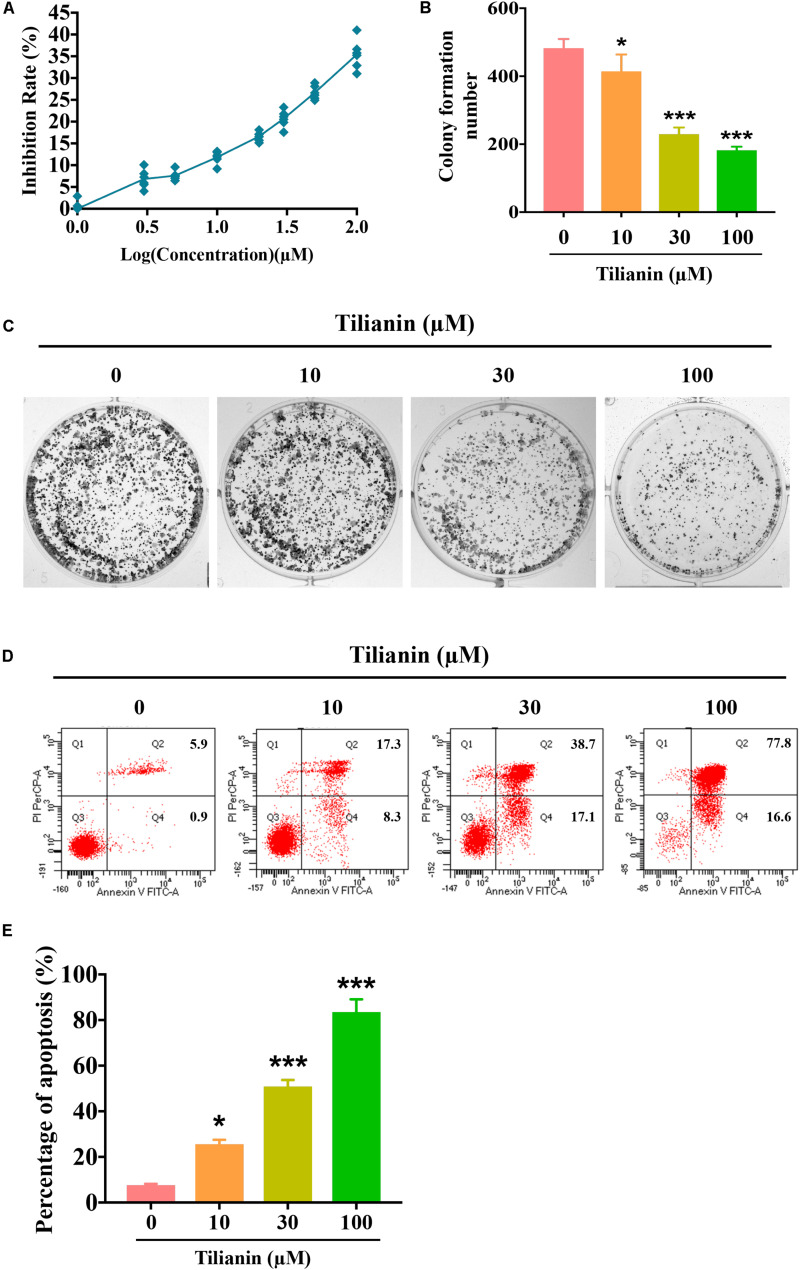
Tilianin inhibits cell proliferation and induces cell apoptosis in FaDu cells. **(A)** Tilianin treatment inhibits cell proliferation of FaDu cells as evaluated by CCK-8 assay after 72 h. **(B)** The numbers of colonies calculated by image J. software. **(C)** Tilianin suppresses plate colony formation of FaDu cells. **(D)** Tilianin induces cell apoptosis in FaDu cells after 24 h as determined by flow cytometry. **(E)** The percentage of apoptosis analyzed by FACSCanto II flow cytometer. Results are presented as the mean ± SD, *n* = 6. * *P* < 0.05, *** *P* < 0.001 vs. control.

To assess the anti-tumor activity of tilianin in FaDu cells, CCK-8 assays, plate cloning formation assays, and Annexin V-FITC/PI staining apoptosis assays were used. The results of the CCK-8 assay showed that treatment with tilianin at 100 μM for 72 h significantly reduced the cell viability of FaDu cells in a concentration-dependent manner (Figure 2A). Correspondingly, in the 14-day culture plate cloning assay, tilianin inhibited colony formation of FaDu cells at 10 —100 μM in a concentration-dependent manner (Figures 2B,C, *P* < 0.05–0.001). Furthermore, tilianin treatment induced apoptosis of FaDu cells, and 10 —100 μM tilianin increased the total apoptosis rate of FaDu cells 3.0 to 4.0-fold, respectively (Figures 2D,E, *P* < 0.05–0.001).

### Intrinsic Apoptotic Signaling Pathways Were Involved in Tilianin-Induced Apoptosis in FaDu Cells

**FIGURE 3:**
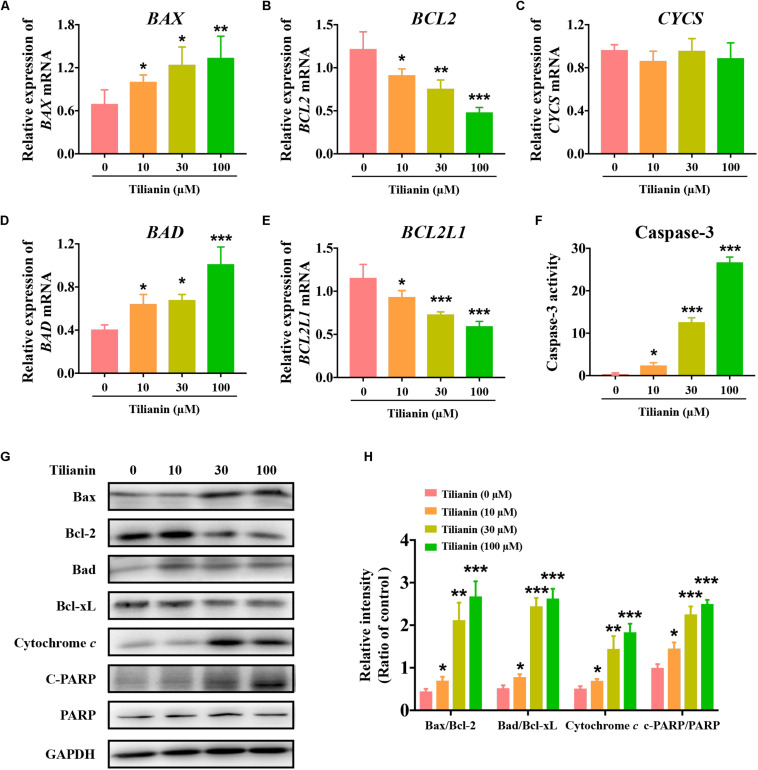
Tilianin induces apoptosis mediated by intrinsic apoptotic signaling pathways in FaDu cells. Quantification of *BAX* **(A)**, *BCL2* **(B)**, *CYCS* **(C)**, *BAD* **(D)**, and *BCL2L1* **(E)** mRNA levels after tilianin treatment. **(F)** Tilianin treatment increases the activity of caspase-3 in FaDu cells. **(G)** Representative Western blot analysis of c-PARP, PARP, Bax, Bcl-2, Bad, Bcl-xL, and cytochrome *c* among groups. **(H)** Quantitative analysis for Western blot analysis shown in **(G)**. Results are presented as the mean ± SD, *n* = 4. * *P* < 0.05, ** *P* < 0.01, *** *P* < 0.001 vs. control.

To elucidate the apoptotic signaling pathways involved in tilianin-induced apoptosis in FaDu cells, both mRNA and protein expression of apoptosis-associated factors were determined by qPCR and Western blot analysis. The data showed that both mRNA and protein expression of the pro-apoptotic factors Bax and Bad were significantly increased in FaDu cells that were treated with tilianin (Figures 3A,D,G,H, *P* < 0.05–0.001). In contrast, treatment with tilianin significantly decreased anti-apoptotic factors Bcl-2 and Bcl-xL in FaDu cells (Figures 3B,E,G,H, *P* < 0.05–0.001). Tilianin increased the protein expression of cytochrome *c* (Figures 3G,H, *P* < 0.05–0.001), but not the mRNA expression (Figure 3C), suggesting that tilianin might stimulate the release of cytochrome *c* from mitochondria, but not affect transcriptional synthesis. Furthermore, active caspase-3 (Figure 3F, *P* < 0.05–0.001) and downstream cleaved- poly ADP ribose polymerase (PARP) (Figures 3G,H, *P* < 0.05–0.001) were significantly up-regulated in FaDu cells that were treated with tilianin. Taken together, these data indicated that tilianin-induced apoptosis of FaDu cells might occur through the mitochondrion-dependent intrinsic apoptotic signaling pathway, ultimately resulting in the activation of caspase-3 and downstream PARP.

### Tilianin Induced DC Maturation

**FIGURE 4:**
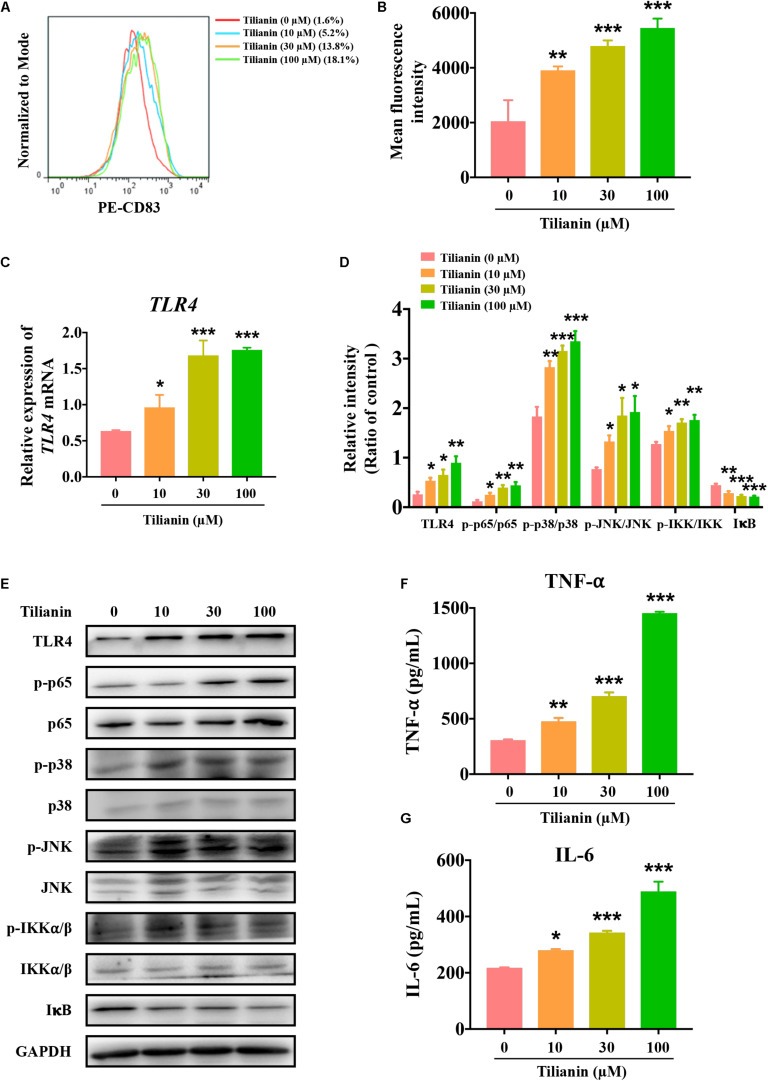
Tilianin induces dendritic cell maturation and activates the TLR4 signaling pathway. **(A)** Representative histograms showing the expression of cell surface dendritic cells (DCs) maturation marker CD83. **(B)** Tilianin increases the mean fluorescence intensity of CD83 of DCs. **(C)** Quantification of *TLR4* mRNA levels after tilianin treatment. **(D)** Quantitative analysis for Western blot analysis shown in **(E)**. **(E)** Representative Western blot analysis of TLR4, p-p65, p65, p-p38, p38, p-JNK, JNK, p-IKKα/β, IKKα/β, and IκB among groups. The level of TNF-α **(F)** and IL-6 **(G)** in cell supernatants measured by ELISA. Results are presented as the mean ± SD, *n* = 4. * *P* < 0.05, ** *P* < 0.01, *** *P* < 0.001 vs. control.

After incubation of immature DCs for 6 days with supernatant of each of the experimental conditions, the degree of DC maturation was determined by flow cytometry. This was mainly done through screening CD11c-positive cells as DCs, and by detecting the proportion of CD83-positive cells in this population. The data showed that, when compared with the control, supernatant from tilianin-treated FaDu cells promoted DC maturation, which was mainly manifested as the increase in the proportion of CD83-positive cells and the fluorescence intensity of CD83 (Figures 4A,B, *P* < 0.01–0.001). Thus, these findings indicated that tilianin may exert the anti-tumor effect by promoting DC maturation, thereby further activating the immune system.

### Tilianin Activated the TLR4 Signaling Pathway

Previous reports have shown that both LPS and TNF-α are classical agents that stimulate DC maturation (Zhou and Tedder, 1996; Michielsen et al., 2012). As shown in Supplementary Figure 2, LPS and TNF-α significantly increased the proportion of CD83-positive cells and the fluorescence intensity of CD83 (Supplementary Figures 2A,B, both *P* < 0.001). LPS is an agonist of the TLR4 signaling pathway, and TNF-α is a cytokine that is secreted by the TLR4 signaling pathway (Zusso et al., 2019). In view of the effect of tilianin on DC maturation as mentioned above, the effect of tilianin on the TLR4 signaling pathway in FaDu cells was evaluated. Our results indicated that tilianin enhanced the expression of TLR4 at both the mRNA and protein level in a dose-dependent manner (Figures 4C–E, *P* < 0.05–0.001). Furthermore, treatment with tilianin promoted the phosphorylation of p65, p38, and JNK, which indicated activation of the TLR4 signaling pathway (Figures 4D,E, *P* < 0.05–0.001). Correspondingly, tilianin promoted the phosphorylation of IKKα/β and the degradation of IκB, thereby indicating the increase in p65 activity (Figures 4D,E, *P* < 0.05–0.001). Finally, tilianin dose-dependently increased the secretion of TNF-α and IL-6 (Figures 4F,G, *P* < 0.05–0.001). Therefore, we hypothesized that tilianin may promote the release of cytokines by activating the TLR4 signaling pathway, thereby stimulating DC maturation.

### TLR4 and NF-κB p65 Mediated Cytotoxic Effects of Tilianin on FaDu Cells Involving Cell Proliferation Inhibition, Apoptosis, and Stimulation of DC Maturation

**FIGURE 5:**
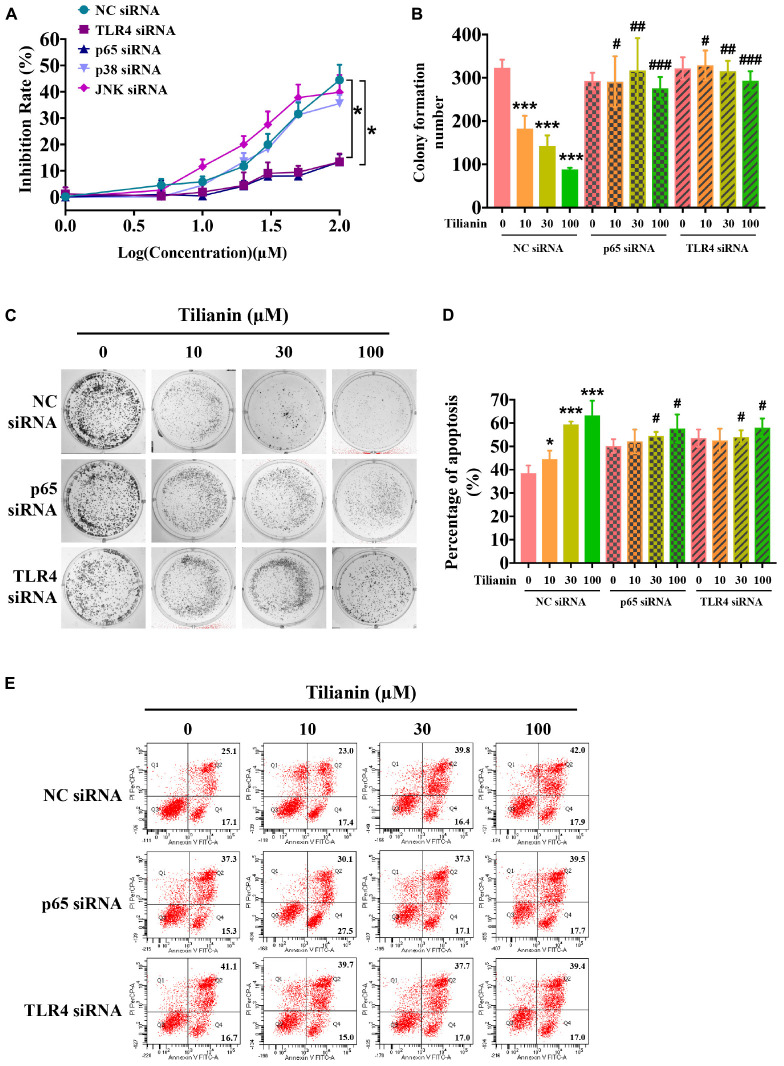
TLR4 and p65 contribute to the cytotoxic effects of tilianin on FaDu cells. **(A)** Tilianin treatment does not decrease cell viability of FaDu cells after silencing of TLR4 and p65 by siRNA. **(B)** Colony numbers calculated by image J. software. **(C)** Tilianin treatment does not inhibit cell colony formation in the presence of TLR4 siRNA and p65 siRNA. **(D)** The percentage of apoptosis analyzed by BD FACSCanto II. **(E)** Tilianin treatment does not induce cell apoptosis after treatment of FaDu cells with TLR4 siRNA and p65 siRNA. Results are expressed as the mean ± SD, *n* = 6. * *P* < 0.05, *** *P* < 0.001 vs. control. ^#^*P* < 0.05, ^##^*P* < 0.01, ^###^*P* < 0.001 vs. tilianin.

To determine whether activation of the TLR4 signaling pathway contributed to tilianin-mediated FaDu cytotoxicity, siRNAs of key components in the TLR4 signaling pathway were used (Supplementary Figures 3A–E, all *P* < 0.05). As shown in Figure 5A, silencing of TLR4 and NF-κB p65 using siRNA prevented the inhibition of proliferation of FaDu cells by tilianin at the concentrations used, whereas p38 siRNA and JNK siRNA blocked the cytotoxic effect of tilianin on FaDu cells (Figure 5A, both *P* < 0.05). Similarly, the inhibitory effect of tilianin on colony formation also disappeared when p38 siRNA and JNK siRNA were transfected (Figures 5B,C, *P* < 0.05–0.001). Thus, these results further indicated that the effect of tilianin on apoptosis of FaDu cells disappeared by the action of these siRNAs (Figures 5D,E, all *P* < 0.05).

**FIGURE 6:**
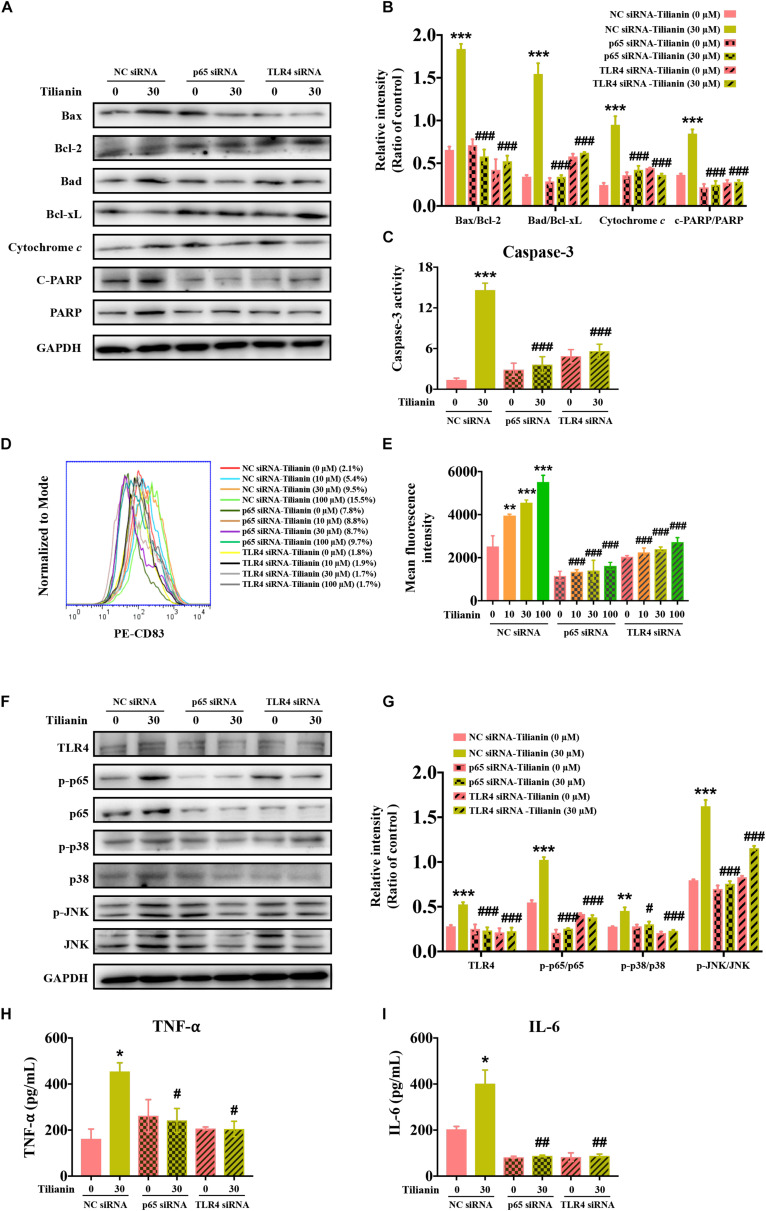
TLR4 and p65 mediate regulatory effects of tilianin on tumor immunity. **(A)** Representative Western blot analysis of c-PARP, PARP, Bax, Bcl-2, Bad, Bcl-xL, and cytochrome *c* among groups. **(B)** Quantitative analysis for Western blot analysis shown in **(B)**. **(C)** The activity of caspase-3 in FaDu cells among groups measured by commercial kit. **(D)** Representative histograms showing the expression of the cell surface dendritic cell (DC) maturation marker CD83. **(E)** Tilianin does not increase the mean fluorescence intensity of CD83 of DCs in the presence of TLR4 siRNA and p65 siRNA. **(F)** Representative Western blot analysis of TLR4, p-p65, p65, p-p38, p38, p-JNK, and JNK among groups. **(G)** Quantitative analyses for Western blot analysis shown in **(F)**. The level of TNF-α **(H)** and IL-6 **(I)** in cell supernatants measured by ELISA. Results are presented as the mean ± SD, *n* = 4. * *P* < 0.05, ** *P* < 0.01, *** *P* < 0.001 vs. control, ^#^*P* < 0.05, ^##^*P* < 0.05, ^###^*P* < 0.001 vs. tilianin.

Next, the expression of biomarkers of the intrinsic apoptotic pathway in FaDu cells were examined using siRNA. The data showed that the effect of 30 μM tilianin on the intrinsic apoptosis signaling pathway of FaDu cells disappeared when using siRNA (Figures 6A–C, all *P* < 0.001), which was consistent with the results obtained by flow cytometry described above. Thus, these results indicated that in FaDu cells, tilianin specifically promoted the intrinsic apoptotic pathways via TLR4 and p65 activation.

Finally, we examined the effect of tilianin on DC maturation as well as changes in the TLR4 signaling pathway following the transfection with siRNA. The results showed that after transfection with siRNA, the stimulatory effect of tilianin on DCs, and activation of the TLR4 signaling pathway disappeared (Figures 6D–G, *P* < 0.05–0.001). In addition, the secretion of downstream proinflammatory cytokines decreased (Figures 6H,I, *P* < 0.05–0.01). Therefore, these findings suggested that TLR4 and p65 may play a key role in the regulation of inflammatory microenvironment responses of the anti-tumor effect of tilianin.

## Discussion

In this study, the cytotoxic effect of tilianin on FaDu cells and its underlying mechanisms involved were elucidated. The results indicated that treatment with tilianin inhibited the proliferation of FaDu cells, induced apoptosis, promoted the release of inflammatory cytokines, and stimulated DC maturation. Moreover, these findings indicated that tilianin treatment up-regulated the expression of the intrinsic apoptotic pathway as well as the TLR4/p65 pathway. More importantly, the cytotoxic effects of tilianin disappeared after silencing of TLR4 and p65 using siRNA. Taken together, these observations indicated that tilianin may exert cytotoxicity and immune surveillance against FaDu cells by acting on TLR4 and p65, thereby providing novel insights into the treatment of human pharyngeal squamous cell carcinoma.

Tilianin is one of the representative active compounds from the plant *D. moldavica*, and is a drug candidate for myocardial ischemia/reperfusion injury therapy, which is supported by the Major Scientific and Technological Special Project for “Significant New Drugs Creation” in China. Using modern pharmacological techniques, the anti-tumor activity and potential underlying mechanisms of tilianin on FaDu pharyngeal squamous carcinoma cells were determined by affecting tumor cell immune escape, which revealed a novel function and indication of tilianin within ethnic medicines.

There is accumulating evidence that tilianin has anti-tumor effects (Meng, 2018; Meng et al., 2018), however, there is an evident controversy about tilianin cytotoxicity in several cancer cell lines, such as HCT116 cells and U87MG cells, which are both sensitive and their resistant counterparts (Nganou et al., 2019). In line with the finding that tilianin possessed cytotoxic activities, in this study, tilianin illustrated to exert cytotoxicity and immune surveillance against FaDu cell survival by acting on TLR4 and p65.

Among the anti-tumor activity of tilianin, 10, 30, and 100 μM of tilianin firstly showed inhibition of the proliferation of FaDu cells within 72 h, and decreased plate colony formation within 14 days, suggesting that tilianin had a cytotoxic effect on FaDu cells. Next, flow cytometry results indicated that tilianin increased the apoptosis ratio of FaDu cells and induced cell apoptosis. Furthermore, tilianin induced apoptosis through the intrinsic apoptosis signaling pathway, including promoting proapoptotic factors Bax and Bad, and down-regulating anti-apoptotic factors Bcl-2 and Bcl-xL. Thus, tilianin had an anti-tumor effect on FaDu cells.

Moreover, to evaluate the effect of tilianin on the immune system, PBMCs were isolated from peripheral blood of healthy individuals and the formation of DCs was induced. It is well-known that the activation state of DCs is more important than the number of DCs, because immature DCs induced tolerance, whereas activated DCs induced immunity against the tumor (Banchereau et al., 2000). In addition, mature DCs have antigen-presenting effects that activated the immune system to clear foreign substances (Wculek et al., 2019). Taken together, these results demonstrated that, when compared with the control group, tilianin increased DC maturation in a dose-dependent manner, indicating that tilianin in part exerted activation effects on immune system.

Furthermore, LPS and TNF-α served as positive controls, and were found to significantly activate DC maturation. Both LPS and TNF-α positively related to the TLR4 signaling pathway (Zusso et al., 2019). Therefore, expression of components of the TLR4 signaling pathway were further examined. Our findings indicated that tilianin activated the TLR4/p65 signaling pathway in FaDu cells, including increased expression of TLR4, the activity of p65, JNK, p38, and promoted the release of downstream inflammatory cytokines, TNF-α and IL-6. Therefore, tilianin was suggested to play a role in activating DC maturation by activating the TLR4/p65 signaling pathway.

In general, TLR4 signaling activates three different pathways, namely, the NF-κB signaling pathway, the p38 MAPK signaling pathway, and the JNK signaling pathway, and promotes the release of proinflammation factors, including TNF-α (Nardo, 2015). Data have shown that TNF-α binds to TNF receptors to activate intrinsic apoptotic pathways in cells, and promote cell apoptosis (Kalliolias and Ivashkiv, 2016). The stress protein JNK and transcription factor p65 also triggered the apoptosis pathways dependent or independent of TNF-α (Liu and Lin, 2005; Dhanasekaran and Reddy, 2008; Pan et al., 2017). Therefore, upregulation of components in the TLR4 signaling pathway can stimulate DC maturation and lead to apoptosis.

The beneficial effects of LPS or a bacterial infection on tumors were investigated in previous studies (Pawelek et al., 2003). Studies have shown that *S. choleraesuis* was used as a tumor killing agent and carrier for tumor-targeted gene therapy (Lee et al., 2008). In mechanistic studies, *S. choleraesuis* was found to cause a strong inflammatory response at the tumor site by acting on TLR4 to recruit a variety of immune cells against tumor cells (Lee et al., 2008). Thus, TLR4 is involved in the regulation of anti-tumor immunity in the tumor-bearing host.

NF-κB p65 has a normal physiological function that mediates an immune response (Zhang and Ghosh, 2001). In the human body, the inflammatory response following an infection requires initiation of the NF-κB signaling pathway, which transcribes several cytokines to mediate an immune response to clear invading pathogens (Beinke and Ley, 2004). However, previous studies have also shown that NF-κB induced chronic inflammation and promoted tumorigenesis (Viatour et al., 2005). Thus, how to balance normal physiological function of NF-κB p65 with chronic inflammation that induced tumors, is the focus of future drug development.

Previous studies have shown that TLR4 and NF-κB p65 were related to the action of tilianin (Wang et al., 2018; Shen et al., 2019). In this study, we aimed to identify the role of tilianin in FaDu cells, using siRNA to target the TLR4 signaling pathway to confirm the tilianin site of action. The results showed that using TLR4 siRNA and p65 siRNA, the cytotoxic effects of tilianin as well as the inhibition of cell colony formation disappeared. Moreover, the apoptosis-inducing effects disappeared, and up-regulation of the intrinsic apoptotic pathway also disappeared. Furthermore, the results showed that TLR4 siRNA and p65 siRNA blocked the maturation of tilianin-stimulated DCs, and activation of the TLR4 signaling pathway by tilianin disappeared. Altogether, the anti-tumor activity, stimulation of DC maturation, and TLR4 signaling activation of tilianin were all blocked by the action of TLR4 siRNA and p65 siRNA, which indicated that tilianin exerted anti-tumor activity and tumor cell immune escape inhibition via TLR4 and p65. Considering the crucial role of TLR4 and p65 in tumor treatment, tilianin may be a potential drug for the treatment of pharyngeal squamous cell carcinoma.

Despite the encouraging results obtained, this study has several limitations. First, as there is a large difference between the external and internal environment in the human body, therefore, the potential proposed treatment of tilianin for pharyngeal squamous cell carcinoma needs to be further explored *in vivo*. Second, although in this study it was confirmed that tilianin had an immunomodulatory effect by enhancing TLR4 signaling pathways, and by exerting anti-tumor effects, in this study, the differential expression of TLR4 signaling pathways in human cancer tissues and adjacent tissues was not been explored. Third, an appropriate positive control usually used in clinical practice should be tested during the investigation of cytotoxic effects of tilianin to obtain a more convincing explanation of its therapeutic effects on pharyngeal squamous cell carcinoma.

## Conclusion

**FIGURE 7:**
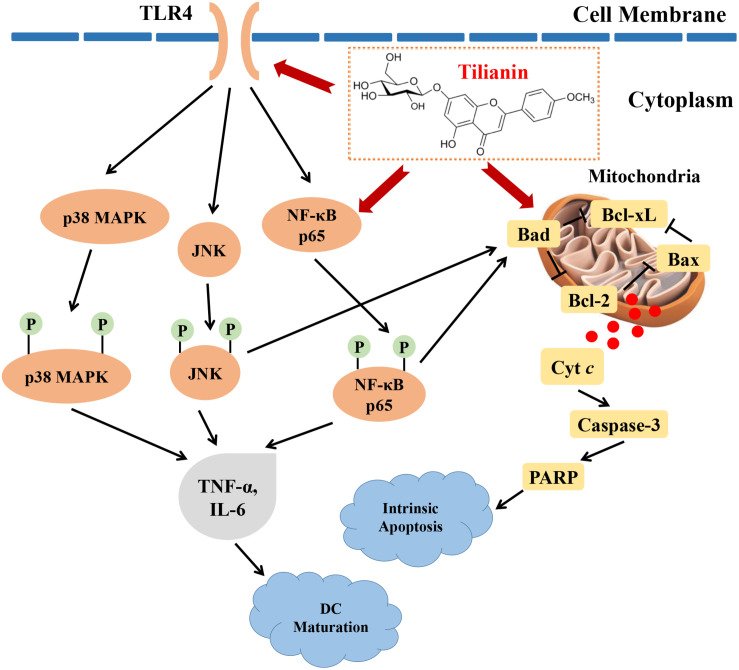
Proposed anti-tumor mechanisms of tilianin on FaDu cells via TLR4 and p65. Tilianin activates the TLR4/p65 signaling pathway and the intrinsic apoptotic pathway by acting on TLR4 and p65 to achieve anti-tumor effects.

In this study, the cytotoxic effects of tilianin treatment on FaDu cells were investigated. Tilianin treatment inhibited the growth of FaDu cells, reduced colony formation, induced apoptosis, and stimulated DC maturation. In addition, tilianin treatment activated the TLR4 signaling pathway, and induced apoptosis via the intrinsic apoptotic pathway (Figure 7). Taken together, these results indicated that tilianin may be a potential therapeutic agent for the treatment of human pharyngeal squamous cell carcinoma.

## Data Availability Statement

The raw data supporting the conclusions of this article will be made available by the authors, without undue reservation, to any qualified researcher.

## Author Contributions

RL and ZL conceived and designed the experiments, acted as principal investigators of the funds and revised and edited the final manuscript. HJ wrote the manuscript. HJ, LZ, XD, and SG performed the experiments and made statistical analysis. HJ and LZ prepared the figures and wrote the manuscript. JX prepared tilianin. All authors read and approved the final manuscript.

## Conflict of Interest

The authors declare that the research was conducted in the absence of any commercial or financial relationships that could be construed as a potential conflict of interest.
