# Corrigendum: Tilianin extracted from *Dracocephalum moldavica* L. induces intrinsic apoptosis and drives inflammatory microenvironment response on pharyngeal squamous carcinoma cells via regulating TLR4 signaling pathways

**DOI:** 10.3389/fphar.2023.1330291

**Published:** 2023-12-01

**Authors:** Hailun Jiang, Li Zeng, Xueqi Dong, Shuilong Guo, Jianguo Xing, Zhuorong Li, Rui Liu

**Affiliations:** ^1^ Institute of Medicinal Biotechnology, Chinese Academy of Medical Sciences and Peking Union Medical College, Beijing, China; ^2^ Department of Cardiology, Fuwai Hospital, National Center for Cardiovascular Disease, Chinese Academy of Medical Sciences and Peking Union Medical College, Beijing, China; ^3^ Department of Gastroenterology, Beijing Friendship Hospital, Capital Medical University, Beijing, China; ^4^ Xinjiang Institute of Materia Medica, Ürümqi, China

**Keywords:** dendritic cells, human pharyngeal squamous cell carcinoma, intrinsic apoptosis, nuclear factor-κappa B, tilianin, toll-like receptor, tumor immunity

In the published article, there was an error in Figure 5 as published. In the originally published version of this article, in Figure 5C, the image for the 10 μM tilianin transfected with NC siRNA group in the cell colony formation assay was incorrect. It inadvertently used the same image as the 100 μM tilianin transfected with p65 siRNA group. The image for the 10 μM tilianin transfected with NC siRNA group in Figure 5C has been corrected with the actual image.

The corrected Figure 5 and its caption appear below.

**FIGURE 5:**
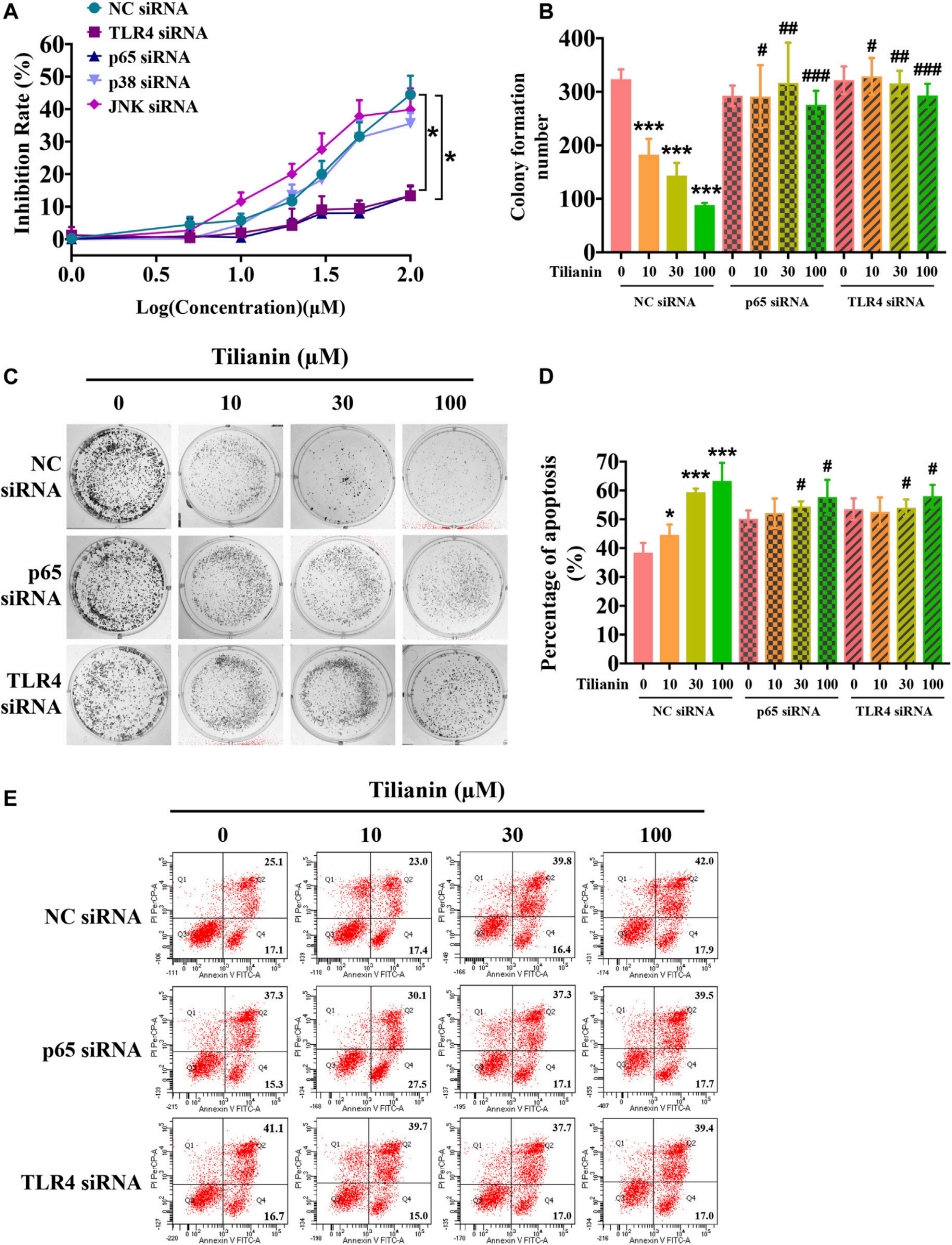
TLR4 and p65 contribute to the cytotoxic effects of tilianin on FaDu cells. **(A)** Tilianin treatment does not decrease cell viability of FaDu cells after silencing of TLR4 and p65 by siRNA. **(B)** Colony numbers calculated by image J. software. **(C)** Tilianin treatment does not inhibit cell colony formation in the presence of TLR4 siRNA and p65 siRNA. **(D)** The percentage of apoptosis analyzed by BD FACSCanto II. **(E)** Tilianin treatment does not induce cell apoptosis after treatment of FaDu cells with TLR4 siRNA and p65 siRNA. Results are expressed as the mean ± SD, *n* = 6. *p < 0.05, ***p < 0.001 vs. control. ^#^ *p* < 0.05, ^##^ *p* < 0.01, ^###^ *p* < 0.001 vs. tilianin.

The authors apologize for this error and state that this change has no impact on the results and conclusions of the article. The original article has been updated.

