# Supplementary material for: Tilianin Extracted From Dracocephalum moldavica L. Induces Intrinsic Apoptosis and Drives Inflammatory Microenvironment Response on Pharyngeal Squamous Carcinoma Cells via Regulating TLR4 Signaling Pathways
Source: Front Pharmacol. 2020 Mar 4;11:205. doi: 10.3389/fphar.2020.00205 (PMC7064631; doi:10.3389/fphar.2020.00205)
Supplement: Supplementary file 1 [file Data_Sheet_1.PDF]

Supplementary Material

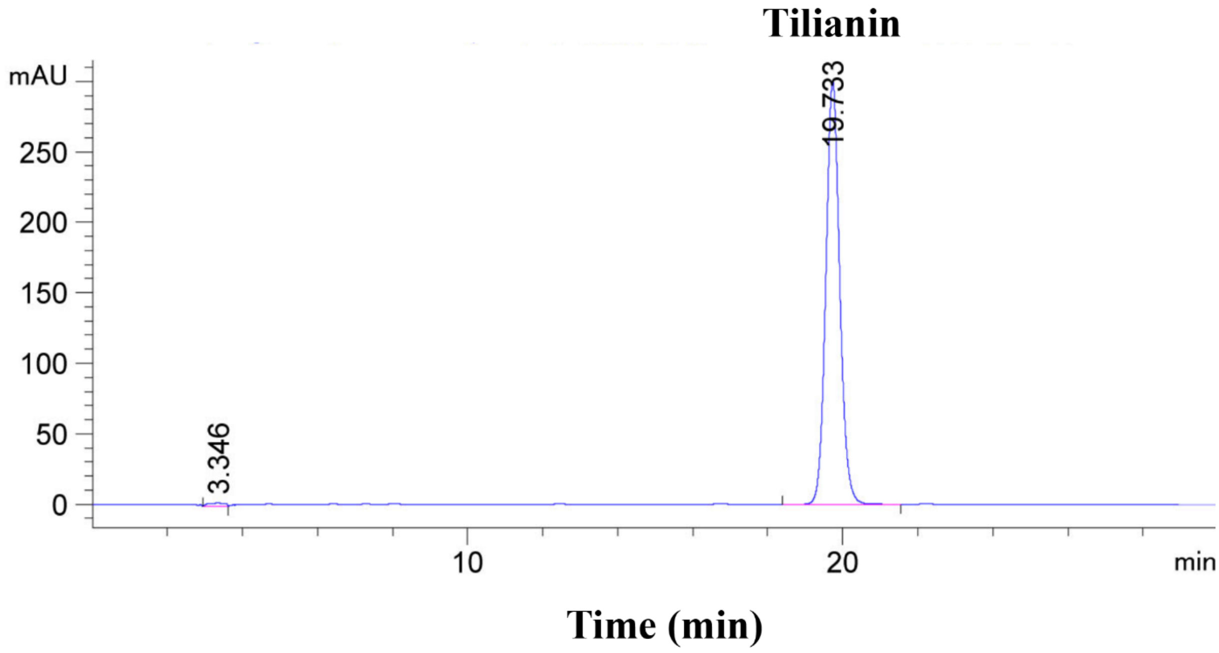

Supplementary Figure 1. HPLC chromatogram of tilianin sample. The purity of tilianin is 99.1211%. The retention time of tilianin peak is 19.733 min.

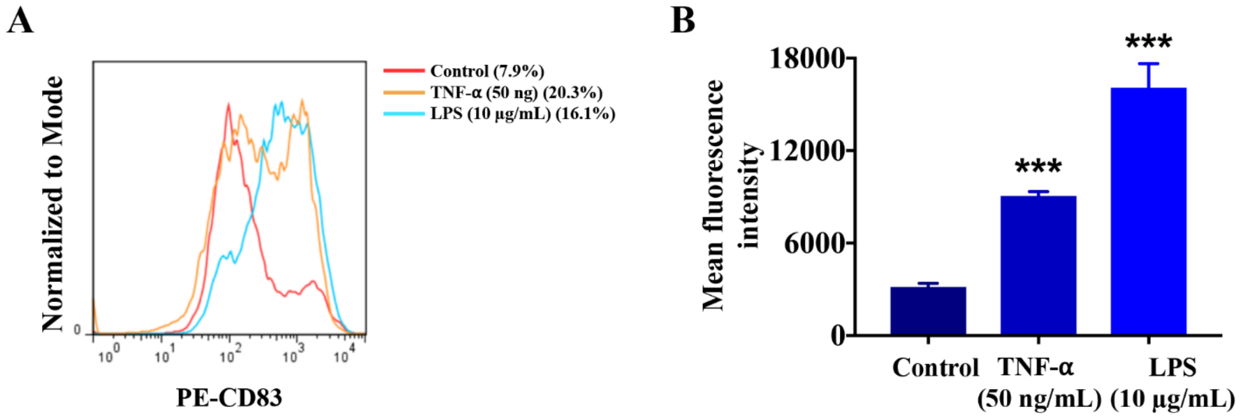

Supplementary Figure 2. LPS and TNF-α induce dendritic cell maturation. (A) Representative histograms showing the expression of the cell surface dendritic cells (DCs) maturation marker CD83. (B) LPS and TNF-α increase the value of mean fluorescence intensity of CD83 of DCs. Results are presented as the mean ± SD, $n = 3$. *** $P < 0.001$ vs. control.

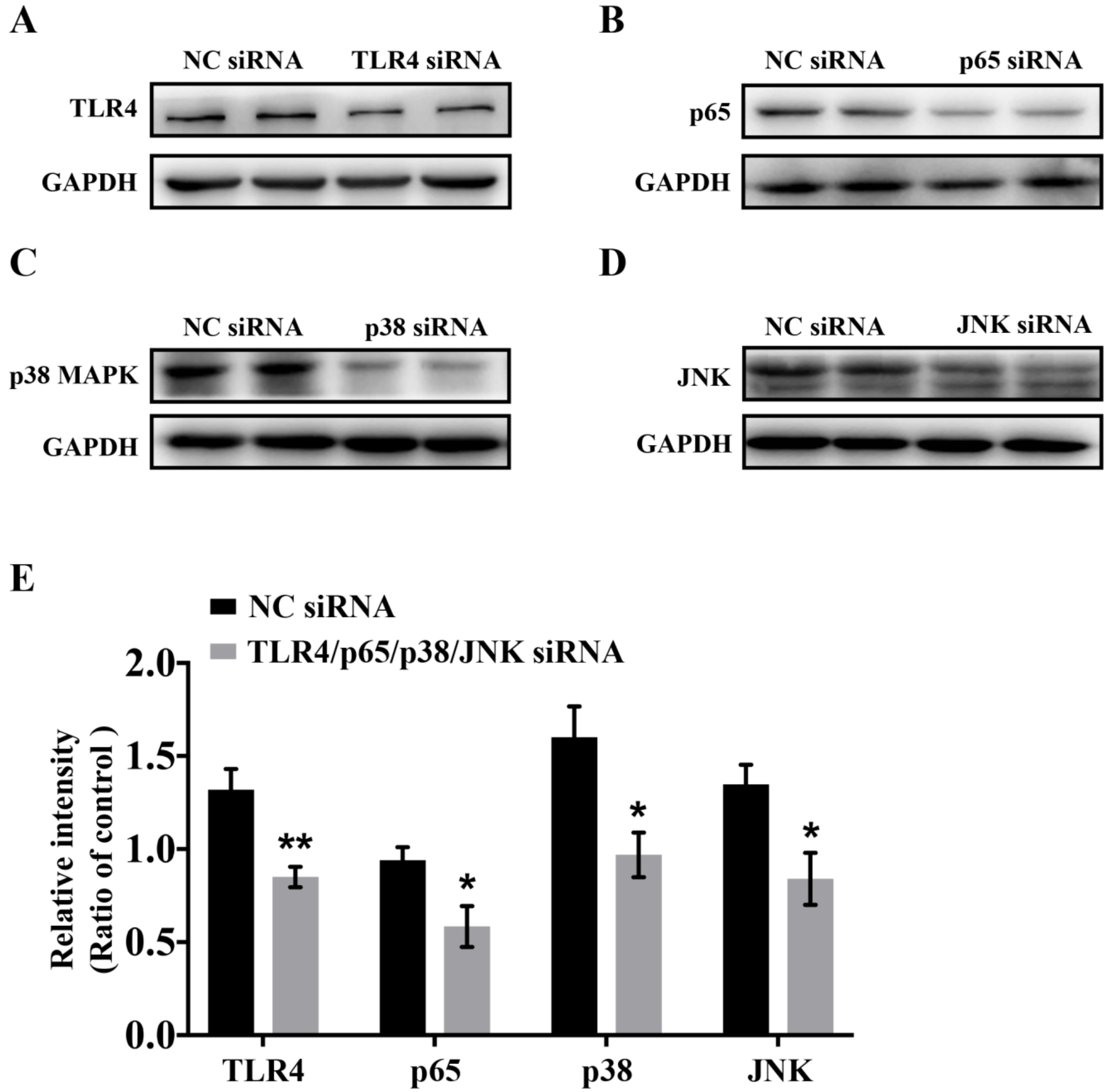

Supplementary Figure 3. The expression of TLR4, NF-κB p65, p38, and JNK after transfection with siRNA. (A-D) Representative Western blot analysis of TLR4 (A), p65 (B), p38 (C), and JNK (D) after transfection with siRNA. (E) Quantitative analyses for Western blot bands of TLR4, p65, p38, and JNK. Results are expressed as the mean ± SD, $n = 3$. *$P < 0.05$, **$P < 0.01$ vs. NC siRNA.
